# Problems of the Grid Size Selection in Differential Box-Counting (DBC) Methods and an Improvement Strategy

**DOI:** 10.3390/e24070977

**Published:** 2022-07-14

**Authors:** Wenxuan Jiang, Yujun Liu, Ji Wang, Rui Li, Xiao Liu, Jian Zhang

**Affiliations:** 1 School of Naval Architecture and Ocean Engineering, Dalian University of Technology, No. 2 Linggong Road, Ganjingzi District, Dalian 116024, China; jwx1991@126.com (W.J.); yjliu@dlut.edu.cn (Y.L.); lirui@dlut.edu.cn (R.L.); liuxiao@dlut.edu.cn (X.L.); 2 Collaborative Innovation Centre for Advanced Ship and Deep-Sea Exploration, Shanghai Jiaotong University, No. 800 Dongchuan Road, Minhang District, Shanghai 200240, China; 3 State Key Laboratory of Structural Analysis for Industrial Equipment, Dalian 116024, China; 4 School of Foreign Languages, Dalian University of Technolog, No. 2 Linggong Road, Ganjingzi District, Dalian 116024, China; janezh@dlut.edu.cn

**Keywords:** fractal dimension, differential box-counting, grid size selection, fractional Brownian motion, Brodatz database, Aerials database

## Abstract

The differential box-counting (DBC) method is useful for determining the fractal dimension of grayscale images. It is simple to learn and implement and has been extensively utilized. However, this approach has several problems, such as over- or undercounting the number of boxes due to inappropriate parameter choices, limiting the calculation accuracy. Many studies have been conducted to increase the algorithm’s computational accuracy by improving the calculating parameters of the differential box-counting method. The grid size is a crucial parameter for the DBC method. Generally, there are two typical ways for selecting the grid size in relevant studies: consecutive integer and divisors of image size. However, both methods for grid size selection are problematic. The consecutive integer method cannot partition the image entirely and will result in the undercounting of boxes; the divisors of image size can partition the image completely. However, this method uses fewer grid sizes to compute fractal dimensions and has a relatively huge distance error (DE). To address the shortcomings of the above-mentioned two approaches, this research presents an improved grid size selection strategy. The improved method enhances computational accuracy by computing the discarded image edge areas in the consecutive integer method, allowing the original image information to be used as thoroughly as the divisor strategy. Based on fractional Brownian motion (FBM), Brodatz, and Aerials image sets, the accuracy of the three grid size selection techniques (consecutive integer method, divisors of image size method, and the improved algorithm) to compute the fractal dimension is then compared. The results reveal that, compared to the two prior techniques, the revised algorithm described in this study minimizes the distance error and increases the accuracy of the fractal dimension computation.

## 1. Introduction

An image captured by a camera is a projection of an actual three-dimensional shape on a plane. The image textures can reflect the morphological features of the physical object. As a result, objects can be identified by evaluating the grayscale variations of an image. Textured surfaces, on the other hand, are inherently complex in natural scenes. Irregular and complex objects cannot be described using Euclidean geometry. Mandelbrot proposed utilizing fractal geometry to describe this type of phenomenon in 1983 [1]. Fractal geometry is used to define self-similar scale-independent elements known as fractal sets. The irregularity of a fractal set can be computed using the fractal dimension. The fractal dimension is now widely utilized in computer vision applications such as texture analysis [2], image segmentation [3], shape recognition [4], pattern recognition [5], time series analysis [6], complex network analysis [7], landslide susceptibility assessment [8], topography analysis of thin films [9], cement-based materials analysis [10], and description of urban morphology [11].

Researchers have proposed many calculating methods to precisely estimate the fractal dimension. Mandelbrot first proposed calculating the fractal dimension when determining the length of the British coastline [1]. Then, based on Mandelbrot’s idea, Pentland [12], Peleg et al. [13], Clarke et al. [14], Dubuc et al. [15], Keller et al. [16], and Gagnepain et al. [17] proposed various methods for determining fractal dimension.

In 1994, Sarkar et al. [18] proposed the differential box-counting (DBC) method to compute the fractal dimension by counting the number of boxes. This method is extensively utilized since it is simple to understand and implement. However, the DBC approach has numerous flaws, including excessive counting of *z*-direction boxes, inappropriate box heights, inaccurate box-counting in the *xy*-direction, and inappropriate box sizes. These weaknesses frequently result in incorrect fractal dimensions, severe distance errors (DEs), and other problems.

For the DBC method, the fractal dimension is calculated by fitting a series of points (log1/r, logN_r_) in a log–log plot. The parameter associated with r is the grid size s, while the N_r_ value is influenced by many parameters, such as the box side length s and height h, the number of boxes n_r_ of each gird, and the partitioning method of the *xy* plane. The calculation methods of these parameters directly impact the calculating accuracy of the DBC method. Many studies have been undertaken to increase the computational accuracy by improving each parameter of the DBC method to tackle the shortcomings of the original DBC approach.

Jin et al. [19] discovered that, during the DBC calculation, the log–log plot presents “steps” under large s values. The central portion of the curve had a relatively constant gradient, while the plot started to level off for smaller values of s. This phenomenon decreased the accuracy of the calculation. Thus, the authors proposed the RDBC approach to lower the DE by optimizing the range of grid size s. Although this study indicates that the trend at both ends of the curve influences the calculation results, it does not give a complete examination of the origins of its phenomenon and does not fix the underlying problems.

Chen et al. [20] presented the SDBC method to overcome the problem of overcounting boxes in the *z*-direction, by introducing a box-shifting mechanism in the *z*-direction to optimize the calculation of n_r_. Li et al. proposed a new n_r_ strategy in 2006 [21] and 2009 [22] to overcome the problem of overcounting boxes in the *z*-direction. They also proposed an overlapping grid partitioning approach to address box over- and undercounting problems along the *xy*-axis, where two nearby grids overlap by one row and one column. However, several shortcomings in this method have been identified in previous literature [23].

Long et al. [24] proposed the integer ratio DBC (IRDBC) to estimate the FD of rectangular images. IRDBC avoided the overcounting of boxes along the *xy*-axis by employing a new n_r_ formula that does not use the ceiling function. As a result, IRDBC generates authentic values for n_r_. However, IRDBC employs only integer values for 1/r, resulting in very few grid sizes being used to compute the fractal dimension.

Liu et al. [25] optimized the formula to compute n_r_ and introduced a grid-shifting mechanism to solve the undercounting of boxes along the *xy*-direction; for non-trivial plane partitioning, the image size divisors were employed as the grid size s. Compared to DBC, they indicated that the improved method enhanced accuracy by 24.1%. Based on Liu’s work, Lai et al. [26] improved the computation of box h. Panigrahy et al. have put in much effort to improve the DBC method. In 2017 [27], they investigated the effect of box height on the accuracy of DBC computation and presented a new method of calculating box height, which considerably enhanced the accuracy of fractal dimension calculation. In 2020 [28], they proposed three new DBC algorithms based on weighted least squares regression. These methods also use a new *xy*-plane shifting mechanism and a modified formula for computing n_r_. However, their method utilized the divisors of image size as the grid size, resulting in a larger DE. In 2021, Liu et al. [29] proposed the IMDBC method, which improved the box-shifting mechanism. The proposed method not only solved the undercounting problem along the *xy*-direction but also had superior stability for image rotation. Compared to DBC and its state-of-the-art methods, the IMDBC method has higher computational accuracy.

According to the studies mentioned above, each algorithm overcomes the shortcomings of the original DBC method by optimizing the calculation parameters to enhance the computing accuracy and minimize distance error. Various parameters affect the computation of the fractal dimension; different approaches have different optimization focuses. Nevertheless, they all achieved better results than the DBC method under their corresponding validation method. However, some studies failed to provide more persuasive evidence on the enhanced accuracy because their validations were not performed using images with known theoretical fractal dimensions, such as synthesized FBM Database [25,29].

Among the various parameters utilized in DBC calculations, the grid size s is crucial. The choice of grid size for a square image impacts how the plane is partitioned during computing. The original DBC approach was based on consecutive integer partitioning, which discards areas that cannot be partitioned in an integer manner [23]. Currently, this strategy is frequently used in studies of state-of-the-art DBC methods [22,26,30,31]. However, in other works, such as those of Liu et al. [25] and Panigrahy et al. [28], the authors utilized a similar approach to that used in Biswas’ study [32], employing divisors of the image size as the grid size s to partition the square image fully.

These two approaches for calculating s values are frequently used in DBC algorithms. Both strategies, however, have limitations. The consecutive integer partitioning method discards the boundary region; hence, the original image is not fully utilized, which will lead to the problem of undercounting boxes. Moreover, as noted previously, the curve of the consecutive integer method is shaped like “steps” at large s values in the log–log plots, which will also affect the calculation accuracy [19]. Although the divisor method can partition the entire image, it omits a significant amount of information at other s values, which leads to result distortion and high DE values [33].

The weight approach has been utilized to compute n_r_ in some methods [23,24,30]. The weight technique is used to calculate n_r_ in a grid whose area is less than s × s and to assign weights based on the actual area of the grid. Long et al. [24] were the first to propose the weight method for calculating the n_r_ values of the boundary grids. Nunsong et al. [30] and Panigrahy et al. [23] then applied the weight technique to the modified triangle box-counting (TBC) methods. In the work of Long et al., the grid partitioning approach is an integer multiple of r values. This strategy would result in fewer s values utilized for calculation and, sometimes, the same s for varied r values [33]. In the studies of Nunsong and Panigrahy, on the other hand, the weight technique was utilized to calculate triangular grids. However, most current DBC algorithms are based on square grids, where the weight method is rarely used. Hence, we propose an improved continuous integer technique for square grid calculation based on the weight method to tackle the problems of the typical divisor method and consecutive integer method.

In order to further investigate the impact of the grid size selection on fractal dimension calculation, this study first introduces the principles of two typical grid size selection methods and assesses their advantages and disadvantages. Then, by processing the discarded edge regions of an image with the weight method, an improved strategy is proposed to tackle the undercounting problem of the original consecutive integer approach and the result distortion problem of the divisor method. Subsequently, based on three image sets (synthesized FBM database, Brodatz database, and Aerials database), the impacts of the three grid size selection methods (original consecutive integer, divisors of image size, and the improved method) were then analyzed, evaluated, and compared. The results reveal that, compared with the original consecutive integer method and the divisor method, the DBC technique based on the improved grid size selection strategy can better estimate fractal dimension values, produce lower DE values, and obtain more consistent goodness of fit values. The various notations and abbreviated terms used in this work are summarized in Abbreviations.

The remaining parts of the paper are as follows: Section 2 describes the classic DBC method, the typical grid size selection strategies, and the drawbacks of each strategy. In the third section, an improved grid size selection strategy based on the consecutive integer method is proposed. Section 4 discusses the three image databases (FBM, Brodatz, Aerials) utilized for validation and evaluation metrics. Section 5 describes and discusses the results of the two typical grid size selection strategies and the improved one based on three image databases. Section 6 presents the conclusions. 

## 2. Materials and Methods

### 2.1. The DBC Method

**Figure 1:**
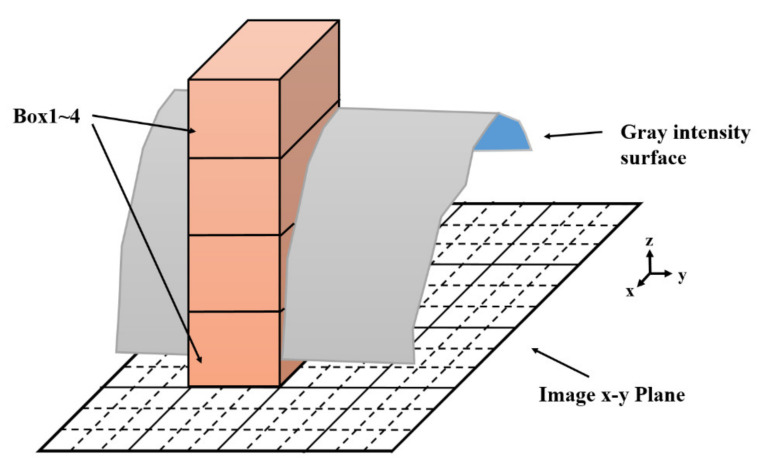
Sketch of determination of the number of boxes (n_r_) by DBC method (here M = 12, s = 3, n_r_ is 3 for the grid).

In 1994, Sarkar et al. [18] proposed a differential box-counting (DBC) technique to compute the fractal dimension of a grayscale image. A square grayscale image with a resolution of M × M can be mapped in 3D space, as illustrated in Figure 1, where the x- and y-directions denote the image length and width. The grayscale values are represented along the *z*-direction. The *xy* plane is divided into non-overlapping grids of size s × s. The values of s vary from 2 to M/2. Non-square grids are formed at the borders if s is not a divisor of M. In this situation, the computation disregards these non-square grids near the boundary. Thus, grids of size s × s partition the plane fully. The grid ratio, r, is calculated using s/M. To represent the gray level variation, n_r_ boxes of size s × s × h are required for each grid, where h is the box height. The following equations are used to compute h and n_r_ (Equations (1) and (2)):

(1) $$\;\lfloor \frac{G}{h}\rfloor =\lfloor \frac{s}{M}\rfloor$$

(2) $$n_{r}\left(i,j\right)=\lceil \frac{g_{\max}}{h}\rceil -\lceil \frac{g_{\min}}{h}\rceil +1$$

G is the total number of grayscale orders in the 8-bit grayscale image, which is 256; g_max_ and g_min_ are the maximum and minimum grayscale values in the (i, j)th grid. Then, the total number of boxes N_r_ corresponding to the ratio r can be calculated using the following formula:

(3) $$N_{r}=\sum_{i,j}n_{r}\left(i,j\right)$$

Finally, the fractal dimension D is computed by fitting the points (log1/r, logN_r_) ∀r with linear least squares regression (LLS). LLS can produce the fitting line y = px + q for these points. The slope and intercept of the fitted line are denoted by p and q, respectively. Here, the slope p is the fractal dimension D that we need.

### 2.2. Two Typical Methods for Determining Grid Size and Their Shortcomings

Many modified DBC methods have emerged based on Sarkar’s DBC method. The grid size s is an essential parameter in these methods. It determines how to partition the *xy* plane. In general, there are two types of grid size selection methods: consecutive integer and divisors of image size. However, each of these solutions has its shortcomings.

#### 2.2.1. Consecutive Integer Method

The original DBC method uses all successive integers between s_min_ and s_max_ as grid sizes. The same partitioning strategy was used in other state-of-the-art methods such as RDBC [19], SDBC [20], Li’2006 DBC [21], Liu’2008 DBC [31], Li’2009 DBC [22], Lai’2016 DBC [26], and Liu’2021 DBC [29]. Most improved algorithms employ this partitioning strategy. Using consecutive integers as grid size can obtain more fitting points for calculating fractal dimensions. However, this method suffers from some limitations. The primary problem with the consecutive integer method is that for large grid size s, several s values correspond to the same N_r_ value, and the relevant curve would be shaped like a “step” in the log–log plot [28].

**Figure 2:**
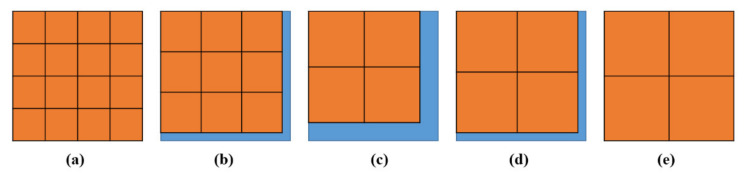
The orange area is the calculated area, and the blue area is the ignored area. Image size is 512 × 512. (**a**) s = 128, (**b**) s = 160, (**c**) s = 220, (**d**) s = 240, (**e**) s = 256.

We take a 512 × 512 image as an example. For the consecutive integer method, s_max_ can be up to 256. At this point, the grids have partitioned the image into four regions (Figure 2e). Several circumstances occur when s is between 128 and 256, as illustrated in Figure 2. When s gradually decreases from 256, the entire image cannot be partitioned completely, and the discarded region (blue) appears, as shown in Figure 2d. Then, s continues to decrease, and the area of the discarded region increases (Figure 2c). Finally, when s is reduced to a value less than 170, as shown in Figure 2b, the number of grids becomes nine, but the plane is still not fully partitioned, even though the discarded area is smaller. When s decreases to 128, as indicated in Figure 2a, the image can be fully partitioned once more.

**Figure 3:**
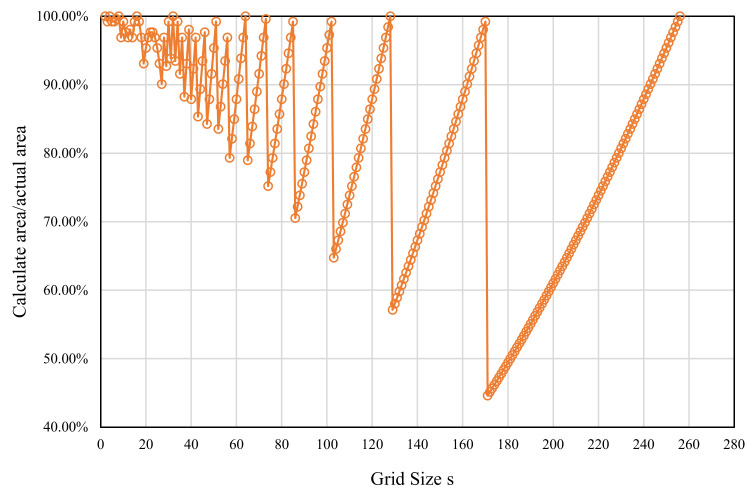
The relationship between the grid size and the percentage of the calculated area to the actual area of the image.

The results in Figure 3 can then be obtained based on the above analysis. The relationship between the actual area for calculation (percentage of the image area) and the gird size s is depicted in Figure 3. The amplitude of the curve increases with increasing s, as shown in the figure. The minimum value of the curve is even less than 50%. When consecutive integers are used as s values, the area used for calculation differs significantly from the actual image area. This deviation becomes more pronounced as the s value increases. Because of the discarded areas, the number of boxes is undercounted, resulting in distorted results.

In theory, a smaller s value should result in a larger N_r_ value. However, due to the vast number of undercounted boxes in this method, especially at large s values, the curve is shaped like a “step”, which is a visual representation of the box undercounting problem.

#### 2.2.2. Divisors of Image Size

Biswas [32] first proposed using $2^{i}|\forall \in Z^{+}$, where $i\in \left[1,\log_{2}\left(M\right)-1\right]$ and $\log_{2}\left(M\right)\in Z^{+}$. Such methods identify the divisors of M to determine s values. This results in grids that partition the entire image without the issue of discarding edge regions, as shown in Figure 2a,e. Some s values, as illustrated in Figure 3, correlate to a calculated area/image area percentage that can reach 100%. In the divisor method, such s values are selected for calculation. This approach is relatively fast since the number of calculated s values is significantly lower than that in the consecutive integer method. All of the s values can completely partition the image, and the corresponding N_r_ values can better capture the properties of the entire image.

However, the reduced number of s values introduces other issues. Images of natural settings are frequently not ideal fractal objects [23] but rather approximate fractals. As a result, fewer computed points may not necessarily represent the image features and cause the results to deviate from the actual fractal dimensions. Moreover, for a 512×512 image, the highest s value of the method is 256, with the second biggest grid size being 128. This significant interval between s values at large scales tends to ignore plenty of grayscale information. Furthermore, the distance error of the divisor method is likewise fairly large [33].

## 3. Improved Consecutive Integer Method

As mentioned above, the divisor method can thoroughly partition the image, but the number of s values is minimal, which can easily lead to distorted results. Although more s values are used in the consecutive integer technique, most of the s values, which cannot entirely partition the image, are prone to the undercounting problem of boxes, especially at large grid size s. Thus, we expect an improved method that can entirely partition the image like the divisor method, but also has numerous s values involved in FD calculation like the consecutive integer method.

**Figure 4:**
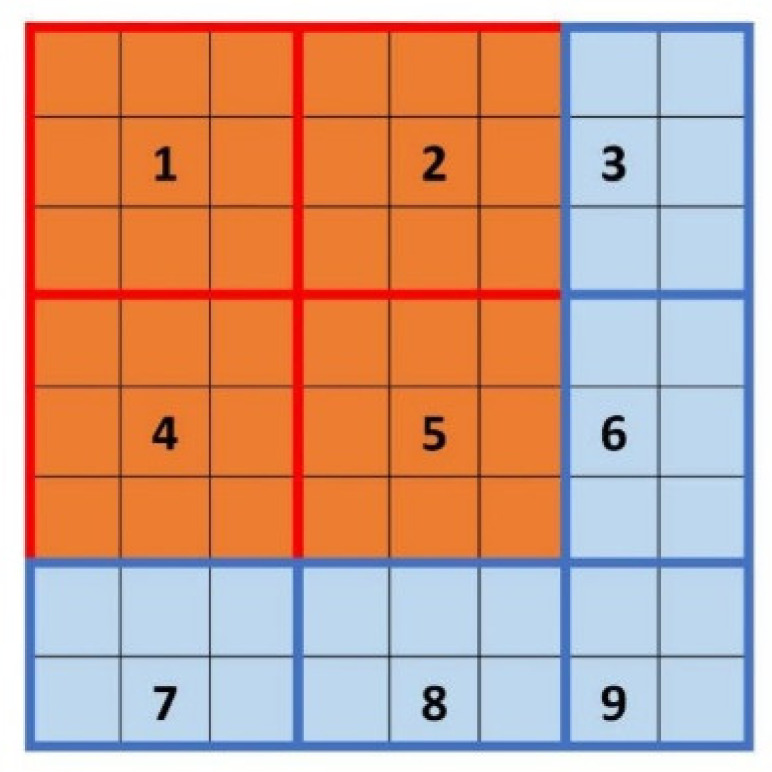
A part of the M×M picture is partitioned by square grids (1, 2, 4, and 5), and the other part cannot be completely partitioned (3, 6, 7, 8, and 9).

To achieve this, we can improve the consecutive integer method by supplementing it with the undercounting boxes. If a given value of s does not completely partition the image, a zone with a width less than s is generated at the boundaries. This is depicted in the blue region of Figure 4. Although the area of each grid in the blue region is less than s × s, it also corresponds to a part of a two-dimensional surface with grayscale variations inside, and the n_r_ value can be computed using Equation (2). However, because the area is less than s × s, the n_r_ value cannot be determined directly as other grids. Thus, the weight approach is introduced here.

The weight method would assign the corresponding weights according to the actual area of the grids [24]. The number of boxes and the area weights need to be considered simultaneously in calculating the n_r_ value of grid (i, j)th. The weight factor F is calculated as shown in Equation (4). A(i, j) is the actual area of the (i, j)th square, and S stands for s × s, which is the area of a grid. F is the ratio of the actual grid area to the theoretical grid area. The corresponding n_r_ value calculation can be obtained from Equation (5).

(4) $$F=\frac{A\left(i,j\right)}{S}$$

(5) $$n_{r}\left(i,j\right)=F\times [\lceil \frac{g_{\max}}{h}\rceil -\lceil \frac{g_{\min}}{h}\rceil +1]$$

**Figure 5:**
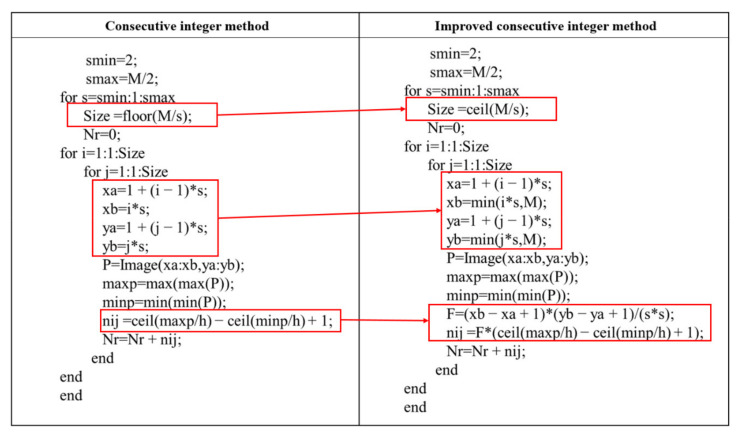
Algorithm code of the DBC method with different consecutive integer methods (**Left**: the original one; **Right**: the improved one).

The code of the DBC method based on two consecutive integer methods is shown in Figure 5. The improved method’s specifics are as follows:

Partition an M×M image into tiny square grids with size s. The number of grids is Size×Size. If M is not divisible by s at this point, non-square regions will form at the margins. As shown in Figure 4, grids 1, 2, 4, and 5 are square regions while grids 3, 6, 7, 8, and 9 are irregular edge regions.

By replacing Equation (2) with Equation (5), the n_r_ value for each grid of the image can be calculated. Subsequently, summing the n_r_ values for each grid gives N_r_ (Equation (6)).

(6) $$N_{r}=\sum_{i,j}n_{r}\left(i,j\right)$$

Finally, linear least squares regression (LLS) is used to fit the obtained points (log1/r, logN_r_) ∀r to calculate the fractal dimension D. Section 5 of the study will further investigate the validity of the method.

## 4. The Experimental Methodology and Evaluation Metrics

### 4.1. Databases for Validation

To compare the performance of the consecutive integer technique, the divisor method, and the improved method, we validate these three approaches using the synthesized FBM images, Brodatz database, and Aerials database. The FBM images can be synthesized based on the theoretical fractal dimension, and the produced grayscale images can be used to validate the accuracy of the three algorithms. The Brodatz and the Aerials databases are typical for validating the fractal dimension algorithm, both obtained in natural scenes. With the help of the Brodatz and the Aerials databases, the algorithms’ performance for texture images and aerial photographs could be evaluated, respectively.

#### 4.1.1. Synthesized FBM Images

In this research, FBM images are synthesized based on the random midpoint displacement (RMD) method. [34] Table 1 tab displays the computational parameters of RMD. The “maxlevel” is used to limit the size of the synthesized image, which is $2^{\mathrm{maxlevel}}+1$; “sigma” is the initial standard deviation; H is the Hurst parameter to determine the actual fractal dimension D = 3 − H. The Boolean parameter “addition” is used to turn on and off the random additions. The “seed” is the seed value for the random number generator.

**Figure 6:**
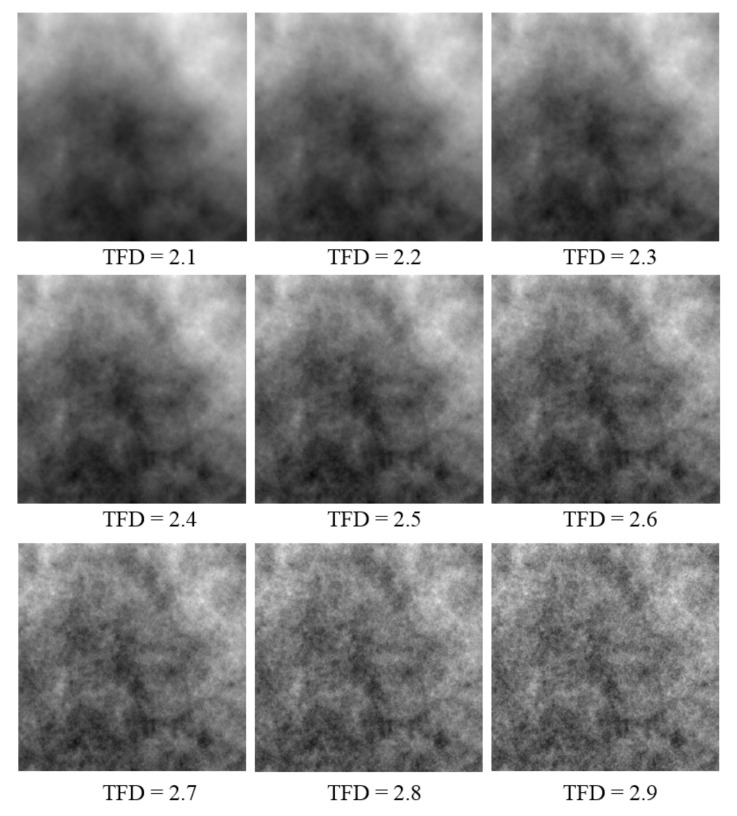
Nine synthesized FBM images with their TFD values.

Nine images with a resolution of 513×513 are created using the RMD method with varied Hurst parameters. To obtain images with a resolution of 512×512, the last row and last column of the image are removed. Figure 6 depicts the synthesized images and their theoretical fractal dimension values.

#### 4.1.2. Brodatz Database

**Figure 7:**
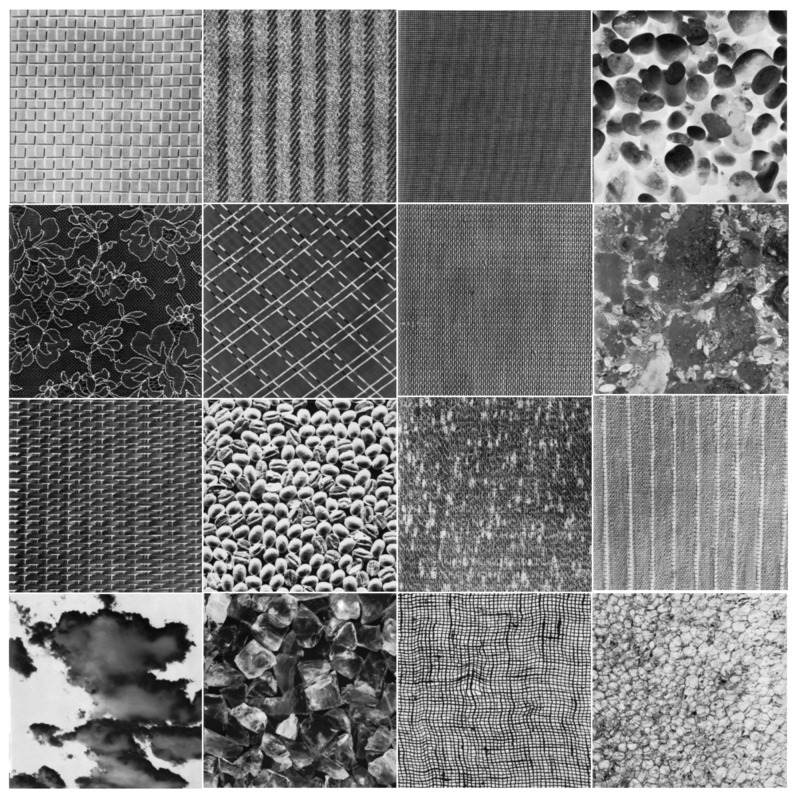
Sixteen sample natural grayscale images of the Brodatz database. The numbering sequence starts from the first row, left to right, top to bottom: D1, D11, D21, D30, D41, D47, D53, D61, D65, D74, D81, D85, D91, D99, D104, D112.

The Brodatz database [35] contains 112 grayscale texture images and is one of the most extensively used image databases. Each image in the database has an unknown theoretical fractal dimension. Sixteen images (shown in Figure 7) were chosen from the database for calculation. Because the images in the Brodatz database are all 640 × 640 resolution, the bicubic algorithm was employed to resize the chosen images to 512 × 512 resolution.

#### 4.1.3. Aerials Database

**Figure 8:**
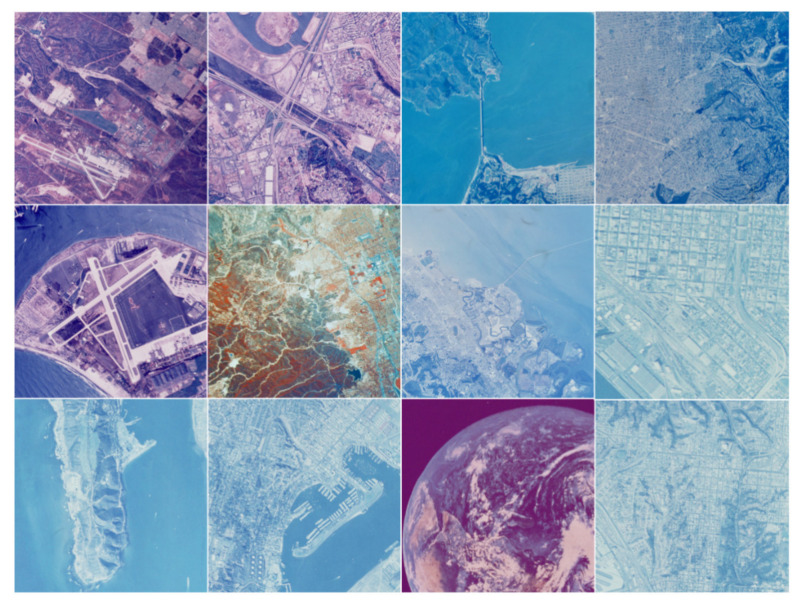
Twelve images of Aerials database. The numbering sequence starts from the first row, left to right, top to bottom: 2.1.01~2.1.12.

As illustrated in Figure 8, twelve satellite images from the SIPI image database [36] were chosen to validate the algorithms’ performance. Because the original photos are not grayscale, they need to be converted into grayscale images before calculation using rgb2gray function in Matlab software. The rgb2gray algorithm used in Matlab is a weighted average of the R, G, and B channels’ values, as shown in Equation (7):

Gaylevel = 0.2989R + 0.5870G + 0.1140B(7)

### 4.2. Evaluation Metrics

Generally, LLS fit to a set of points in a log–log plot is required to calculate the fractal dimension. The goodness of fit (R^2^) measures how well the fitting line matches the scattered points, whereas the distance error (DE) measures the average error between the fitted and actual values. All three databases are calculated using these two assessment measures. The correlation coefficient and slope$\;\hat{b}$ are introduced to the calculation of synthetic FBM images. These two metrics can evaluate whether the estimated fractal dimensions correlate well with the theoretical fractal dimensions.

#### 4.2.1. Goodness of Fit (R^2^)

The goodness of fit is used to evaluate the fit of the regression line to the observed values. The maximum value of R^2^ is 1. R^2^ close to 1 indicates the excellent fit of the regression line to the observed values; conversely, R^2^ close to 0 indicates the poor fit of the regression line to the observed values. For m samples, (x_1_, y_1_), (x_2_, y_2_),…, (x_m_, y_m_), the estimates corresponding to the model are (x_1_, $\hat{y_{1}}$), (x_2_, $\hat{y_{2}}$),…, (x_m_, $\hat{y_{m}}$).

Then, calculate the total sum of squares (TSS):

(8) $$\mathrm{TSS}=\sum_{i=1}^{m}{\left(y_{i}-\;\bar{y}\right)}^{2}$$

Calculate the residual sum of squares (RSS):

(9) $$\mathrm{RSS}=\sum_{i=1}^{m}{\left(\hat{y_{i}}-y_{i}\right)}^{2}$$

Then, we can get:

(10) $$R^{2}=1-\mathrm{RSS}/\mathrm{TSS}$$

#### 4.2.2. Distance Error (DE)

The fractal dimension is calculated using LLS fitting in the various DBC techniques. The distance error (DE) is defined as the root-mean-square distance between the scatter points and the fitted line in a log–log plot. DE is a crucial metric for evaluating DBC methods and has been utilized in many studies [23,29,33]. Various optimizations of the DBC method are also aimed at smaller DE. The calculation equation is shown below.

(11) $$\mathrm{DE}=\frac{1}{m}\sqrt{\frac{\sum_{i=1}^{m}{\left({\mathrm{px}}_{i}+q-y_{i}\right)}^{2}}{1+p^{2}}}$$

#### 4.2.3. Correlation Coefficient γ

Correlation coefficients are used to show the linear relationship between two sets of data. The algorithms provide a number between −1 and 1, where 1 represents a strong positive relationship, −1 represents a strong negative association, and zero represents no relationship at all. For two sets of variables, X(x_1_,x_2_,…,x_m_) and Y(y_1_,y_2_,…,y_m_), the correlation coefficient γ can be calculated by the following equation:

(12) $$\gamma =\mathrm{Correl}\left(X,Y\right)=\frac{\sum \left(x_{i}-\;\bar{x}\right)\left(y_{i}-\;\bar{y}\right)}{\sqrt{\sum {\left(x_{i}-\;\bar{x}\right)}^{2}\sum {\left(y_{i}-\;\bar{y}\right)}^{2}}}$$

#### 4.2.4. Slope $\;\hat{b}$

$\;\hat{b}$ is the slope of LLS fitting line for m sets of samples (x_1_, y_1_), (x_2_, y_2_),…, (x_m_, y_m_). Corresponding to the model estimates (x_1_, $\hat{y_{1}}$), (x_2_, $\hat{y_{2}}$),…, (x_m_, $\hat{y_{m}}$), the slope b is determined as shown in Equation (13). This metric is utilized in the FBM database calculation to assess how well the fractal dimension calculated by DBC methods matches the theoretical values.

(13) $$\;\hat{b}=\frac{\sum_{i=1}^{m}\left(x_{i}-\;\bar{x}\right)\left(y_{i}-\;\bar{y}\right)}{\sum_{i=1}^{m}{\left(x_{i}-\;\bar{x}\right)}^{2}}=\frac{\sum_{i=1}^{m}x_{i}y_{i}-n\;\bar{x}\;\bar{y}}{\sum_{i=1}^{m}x_{i}^{2}-{n\;\bar{x}}^{2}}$$

### 4.3. Flow Chart of the Experiment

**Figure 9:**
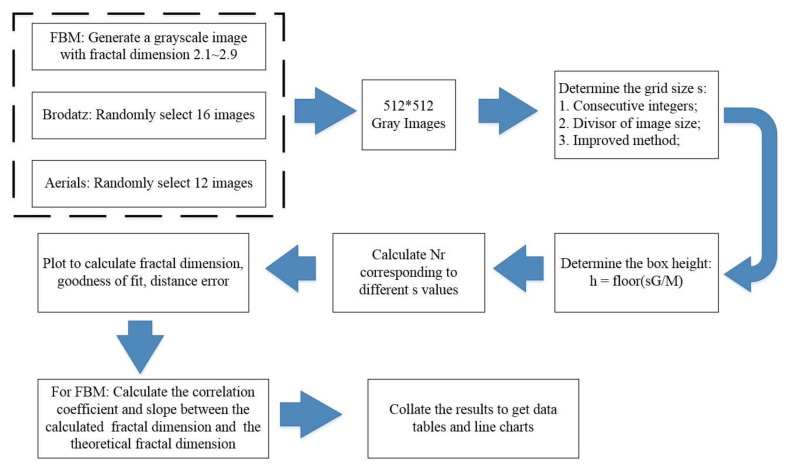
Computational flow chart for experimental research.

Combining the above content, we can get the experimental process of this study, as shown in Figure 9. The experimental results in the fifth section are obtained according to this procedure.

## 5. Results and Discussions

### 5.1. The Effect of Grid Size Selection on the Fractal Dimension Calculation for Synthesized FBM Images

**Figure 10:**
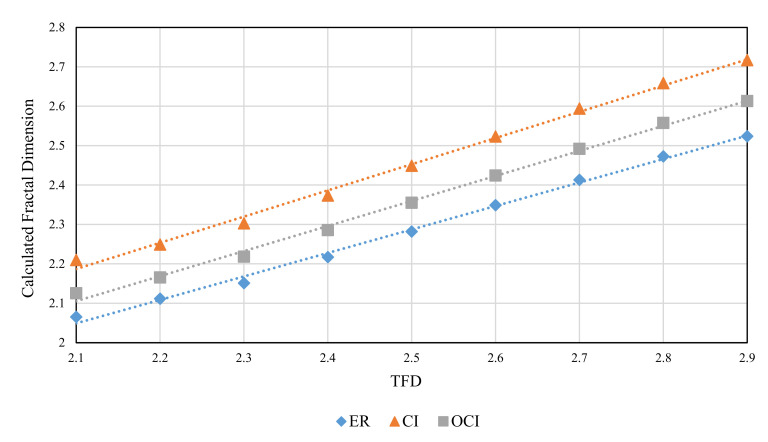
Calculated fractal dimension of images with different theoretical fractal dimension values (FBM).

**Figure 11:**
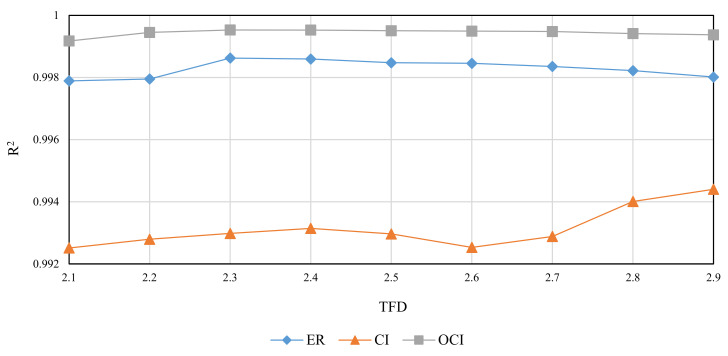
Goodness of fit of images with different theoretical fractal dimension values (FBM).

**Figure 12:**
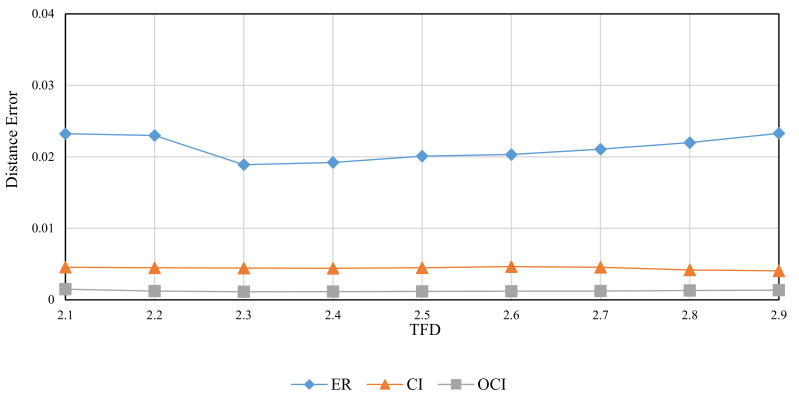
Distance error (DE) of images with different theoretical fractal dimension values (FBM).

The RMD method was used to generate FBM images with theoretical fractal dimensions ranging from 2.1 to 2.9. The following methods were used to calculate grid size: divisor of image size (ER), original consecutive integer method (CI), and optimized consecutive integer method (OCI). Table 2 displays the fractal dimension calculation, the goodness of fit, and the distance error corresponding to the three methods. Figure 10, Figure 11 and Figure 12 illustrate the data in Table 2. The correlation coefficients and slope$\;\hat{b}$ between the theoretical fractal dimensions and the calculated fractal dimensions are shown in Table 3. Theoretically, the estimated fractal dimensions should be identical to the theoretical values, but this does not happen due to methodology error. When the correlation coefficient and slope are near 1, we can conclude that the corresponding method performs well.

Figure 10 shows that the fractal dimension value calculated by the three methods rises as the TFD value increases. The consecutive integer method achieves the largest value from the fractal dimension value calculation, the divisor method produces the smallest value, and the computed value of the enhanced consecutive integer method is between the two. According to the data in Table 3, the improved approach has the largest correlation coefficient, the original consecutive integer method has the smallest, and the divisor method has a correlation coefficient between the two. This demonstrates that the fractal dimension values calculated by the improved consecutive integer method correlate better with TFD values than those of the other two methods. In terms of slope, the improved consecutive integer method has a somewhat lower slope than the original method. However, both have higher slope values than the divisor method. The above results demonstrated that, compared to the consecutive integer methods, the computed fractal dimensions derived by the divisor method have a relatively good correlation with the TFD values but the trend deviation is large. The calculated results of the original consecutive integer method can better match the trend of the TFD values, but the correlation is the worst. The improved method outperforms the other two in terms of linear correlation between calculated FD and TFD values, although it has slightly lower slope values than the original consecutive integer method.

When comparing the goodness of fit results in Table 2 and Figure 11, it can be observed that the improved method has the highest goodness of fit, followed by the divisor method, and the original consecutive integer method has the lowest goodness of fit. When the DE values are compared (Figure 12), the divisor method has the largest distance error; the consecutive integer method has a relatively moderate distance error, and the enhanced method has the smallest distance error.

**Figure 13:**
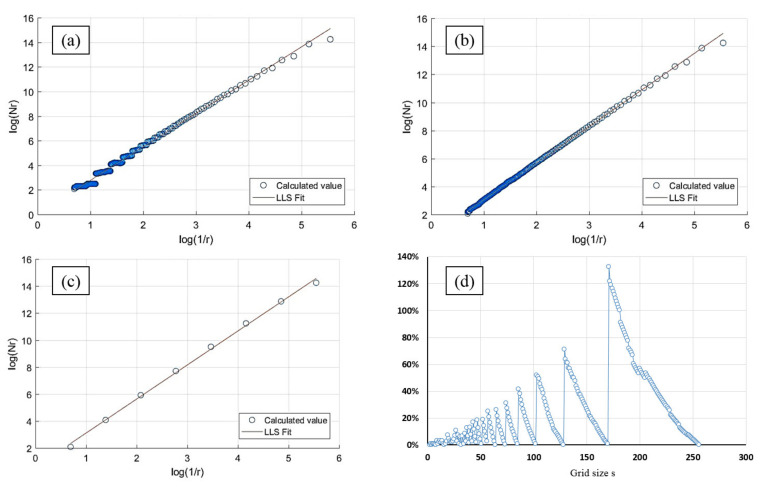
LLS fitting lines of FBM images with a theoretical fractal dimension of 2.9 by three methods. (**a**) Consecutive integer; (**b**) improved consecutive integer method; (**c**) image size divisor; (**d**) compared with the original consecutive integer method, the improved method has an increase in the number of boxes under different s values.

To investigate the causes of this phenomenon, we generated Figure 13 based on the calculation of the FBM image with a theoretical fractal dimension of 2.9. Figure 13a–c depict the log–log plot curves of the three approaches, respectively. We produced Figure 13d to compare the difference in the number of boxes between the improved consecutive integer method and the original method.

In this work, eight s values are employed for the divisor method and 255 for the consecutive integer method. These 255 s values contain the divisor method’s eight s values. This is equivalent to the consecutive integer technique having 247 more data points than the divisor method for LLS fitting. The absence of these 247 points is also responsible for the distinction between the divisor method and the consecutive integer method.

According to Figure 13c, the divisor method requires fewer scatter points for the calculation, which is related to the lower number of s values needed for the calculation. Fewer points reduce the computing work accordingly. However, the relatively small number of grid size values leads to the insufficient utilization of the original image, which can easily distort the fractal dimension result. As a result, although the divisor method has a higher R^2^ value and the obtained fractal dimension value has a higher correlation with the TFD value, the calculated fractal dimensions are smaller than those of the two consecutive integer methods, the distance errors are larger, and the trend deviation of the calculated result from the TFD value is relatively large.

For the consecutive integer methods, more s values are used, and more corresponding N_r_ values are acquired. Even though the calculation amount is substantially larger than that of the divisor method, the grayscale information offered by the original image can be fully utilized. As a result, consecutive integer methods produce higher FD and lower distance error compared to the divisor method, and the computed fractal dimensions trend is closer to the TFD values. However, because the edge region is discarded in the original consecutive integer technique, the undercounting of boxes emerges at most s values. The “steps” in the curve, as seen in Figure 13a, best represent this problem. The improved method reconsiders the discarded boxes and produces a smoother curve, as seen in Figure 13b.

The improved method considerably improves the number of computed boxes compared to the original consecutive integer method, as shown in Figure 13d. The higher the s value, the greater the percentage of improvement. This curve resembles the trend in Figure 3. It can be interpreted as the improved approach compensating exactly for the original method’s discarded boxes, and the larger the s value, the larger the percentage of compensation. Compared to the original consecutive integer method, the improved method makes better use of the image’s grayscale information. The compensating for the discarded boxes, on the one hand, raises the N_r_ values under large s values, which results in a reduction in the slope of the LLS line (i.e., the fractal dimension value) in Figure 13a. As a result, the fractal dimension of the improved method appears to be less than that of the original consecutive integer methods, as illustrated in Figure 10.

On the other hand, the “steps” in the curve of the original consecutive integer method are deleted, the incorrect box-counting is revised, and the computational accuracy is enhanced. Thus, compared to the original consecutive integer method, the improved method achieves larger goodness of fit and a smaller distance error, and the computed fractal dimension has a better correlation with the TFD value.

In summary, the improved consecutive integer method outperforms the divisor method and the original consecutive integer method in computing accuracy for the fractal dimension calculation of FBM images.

### 5.2. The Effect of Grid Size Selection on the Fractal Dimension Calculation for Brodatz Images

**Figure 14:**
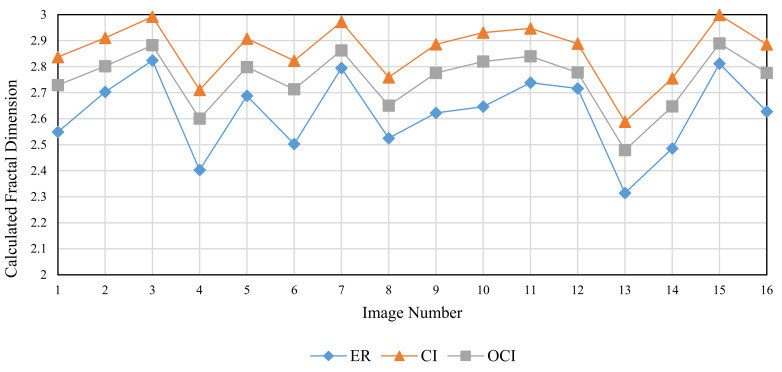
Calculated fractal dimension of different images (Brodatz).

**Figure 15:**
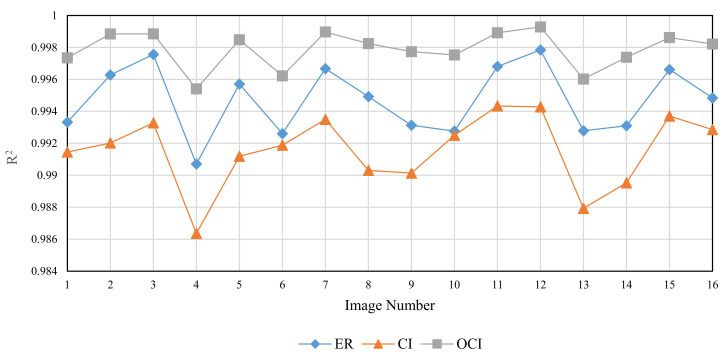
Goodness of fit of different images (Brodatz).

**Figure 16:**
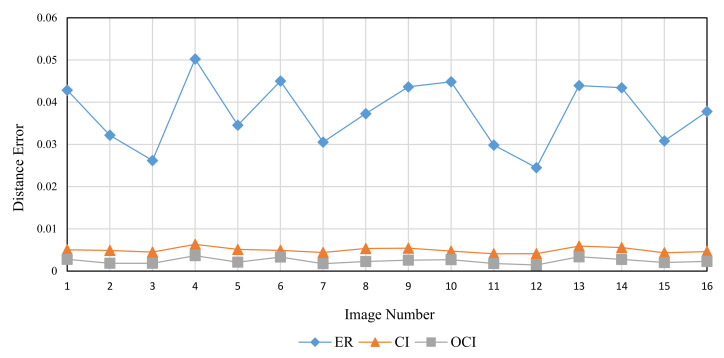
Distance error (DE) of different images (Brodatz).

The Brodatz texture database, a commonly used database in DBC investigations, is utilized for validation to examine the three methods’ performance in fractal dimension calculation of texture images. This database is composed of grayscale images with various textures. The calculation in this section is based on the consecutive integer method, the divisor method, and the improved consecutive integer method for 16 images from the Brodatz database. Table 4 displays the acquired results for fractal dimension, the goodness of fit, and distance error, and Figure 14, Figure 15 and Figure 16 illustrate the data in the table.

Figure 14 shows that the three methods for calculating the fractal dimension have a similar variation trend. The trend depicted in Figure 14 is generally consistent with the prior study [33]. The fractal dimensions acquired by the consecutive integer method are large, while the fractal dimensions produced by the divisor method are small; the fractal dimension result of the improved method is between the two. The improved consecutive integer method has a much higher goodness of fit than the original consecutive integer method, according to the goodness of fit curve in Figure 15; the goodness of fit achieved by the divisor method is between the two. Figure 16 illustrates that the distance error values of the divisor method are higher than those of the two consecutive integer methods, and the improved consecutive integer method obtains the smallest distance errors.

The Brodatz texture database, unlike the FBM images, is derived from the natural scene. As discussed in Section 2.2.2, images of natural scenes are not always ideal fractal patterns [23]. This is where the Brodatz database images differ from the FBM-generated images. It also emphasizes the significance of validating with the Brodatz database. Based on the analysis of the previous section, in the calculation of Brodatz images, the improved consecutive integer method considers edge regions and calculates more boxes than the original consecutive integer method. It is equivalent to raising the value of N_r_ under certain s values, particularly when s is large. Compared to the original consecutive integer approach, the improved method decreases the slope of the fitted line, i.e., the fractal dimension value. For calculating the fractal dimension of texture images, the improved method obtains larger goodness of fit and a smaller distance error compared to the original method. It demonstrates that the conclusions obtained in the previous section are still applicable to texture image calculation.

The preceding section mentioned that using fewer s values can result in distorted computing results for the divisor method. For the Brodatz database, the distortion of the divisor method results in small fractal dimensions and large distance errors. This result agrees with the FBM-based study described above.

In summary, the improved consecutive integer method outperforms the original method for texture image calculation. Because of distortion, the divisor method does not produce satisfactory results when calculating texture images. In 2021, Liu et al. [29] compared their method to the algorithms proposed by Panigrahy et al. in 2020 [28] and Li et al. in 2014 [25]. For the Brodatz database, the mean value of DE calculated by these methods is more than 0.0025. In this study, the Brodatz database’s mean DE value determined by using the DBC algorithm only modified with the improved consecutive integer method is 0.024. Furthermore, only the grid size selection method is optimized in this study compared to the original DBC approach; no box-shifting mechanism or box height change is utilized. This also emphasizes the significance of grid size selection.

### 5.3. The Effect of Grid Size Selection on the Fractal Dimension Calculation for Aerials Images

**Figure 17:**
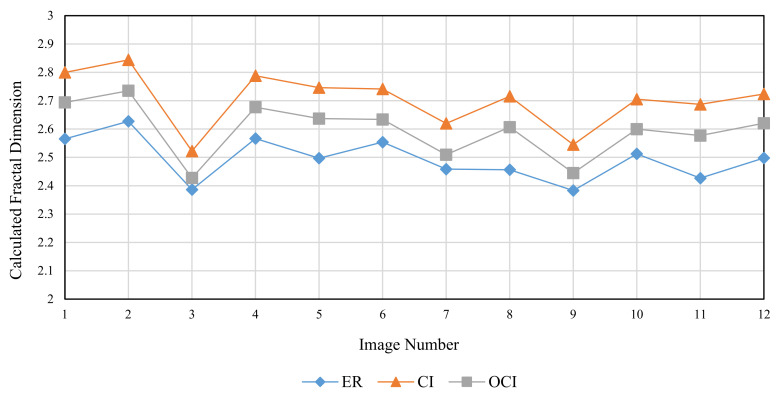
Calculated fractal dimension of different images (Aerials).

**Figure 18:**
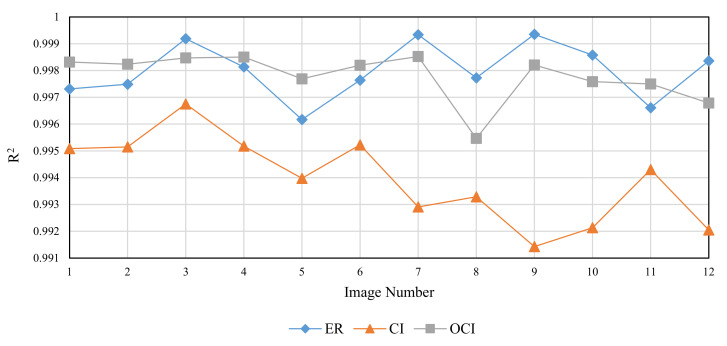
Goodness of fit of different images (Aerials).

**Figure 19:**
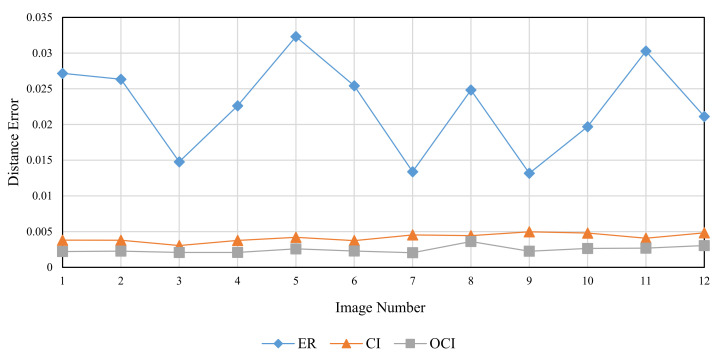
Distance error (DE) of different images (Aerials).

To compare the performance of the three methods, twelve images from the Aerials database with a resolution of 512 × 512 were chosen. As these are high-altitude aerial images, they are suitable for remote sensing applications. Figure 17, Figure 18 and Figure 19 and Table 5 illustrate the fractal dimension, goodness of fit, and DE values obtained by three different methods.

According to Figure 17, the estimated fractal dimensions are identical to those of Brodatz and FBM. The original consecutive integer method produces the highest fractal dimension, the divisor method produces the smallest fractal dimension, and the improved consecutive integer method produces values in between. In terms of goodness of fit, the improved method outperforms the original consecutive integer method. Unlike Brodatz and FBM, the divisor technique’s goodness of fit for Aerials images is almost equivalent to that of the improved consecutive integer approach. The distance error results are similar to the FBM and Brodatz results, with the divisor method generating the highest error, followed by the original consecutive integer method; the improved consecutive integer method produces the lowest error.

The Aerials images are pictures of natural scenes, which are also not ideal fractals. Compared to Figure 14 and Figure 16 in the preceding section, the fractal dimension and distance error results of the Aerials images (Figure 17 and Figure 19) are extremely similar to the computed results of Brodatz images. This also validates the conclusion of the previous section. It indicates that, for Aerials images, the improved method still achieves lower distance errors than the divisor method and the original consecutive integer method.

However, unlike the results for Brodatz images, the goodness of fit values obtained by the divisor method are not intermediate between the two consecutive integer methods. We indicated in Section 5.1 that the divisor method requires eight points to fit an LLS line, whereas the consecutive integer method takes 255 points, including the 8 points of the divisor method and the other 247 data points. The absence of 247 points in the divisor method makes the calculation results “distorted”. The effect of the “distortion” varies depending on the image type. Comparing Figure 7 and Figure 8, it can be seen that the aerial image has different characteristics from the texture image. The Brodatz texture image is more homogeneous and has basically the same features in all image regions, but the Aerials image usually appears with some conspicuous objects.

Because there are fewer s values in the divisor technique, the fitting results will be considerably influenced if the image’s conspicuous objects influence certain s values. Because the consecutive integer method uses a large number of s values, some abnormal scatter points do not affect the results. As a result, in Figure 18, the divisor method’s goodness of fit curve seems to oscillate around the curve of the improved consecutive integer method. Of course, further research is required to confirm this. In summary, the improved consecutive integer method outperforms the divisor method and the original continuous integer method for computing the fractal dimension of Aerials images.

### 5.4. Applying Improved Grid Selection Strategies to Other DBC Methods

From the above analysis based on the original DBC method, it is found that the improved grid selection strategy, i.e., the improved consecutive integer method, can obtain better accuracy of fractal dimension calculation. For further validation, we tested the effectiveness of the improved strategy based on the three methods of Long’2013DBC [24], Lai’2016DBC [26], and Liu’2021DBC [29]. The image database used for the calculation is the same as that described in Section 5.1. The results of the three methods (with/without the improved strategy) are calculated separately based on the FBM images. The obtained results of fractal dimension, goodness of fit, and distance error are shown in Table 6, Table 7 and Table 8.

From the fractal dimension results in Table 6, we can find that the Long’2013 method with the improved strategy obtains a higher fractal dimension than the original method. FDs of Lai’2016 and Liu’2021 methods show a fractal dimension decrease after applying the improved strategy. From the results in Table 7 and Table 8, it can be found that for all three methods, the improved strategy improves the goodness of fit and reduces the distance error compared to the original methods.

The strategy of integer r used in the Long’2013 method leads to fewer values of s involved in the calculation. This is very similar to the divisor method. Therefore, it is easy to produce the problem of small fractal dimension, large goodness of fit, and large distance error. However, because the weight method is used in Long’s method, the distance error obtained is not much different from that of the consecutive integer method even though the above problems exist. After applying the “consecutive integer method + weighting method” strategy, Long’s method could involve more s values and fully use the grayscale variation of the original image, resulting in a larger fractal dimension, higher goodness of fit, and smaller distance error.

In both Lai’2016 and Liu’2021 methods, the original consecutive integer method is used as the grid selection strategy. Thus, even though various optimizations have been performed in their methods, the problems caused by the original consecutive integer method still exist. Based on the analysis of previous sections, the original consecutive integer method has the problem of undercounting boxes. The larger the value of s, the higher the percentage of undercounting boxes. After applying the improved strategy, it is equivalent to raising the vertical coordinates of the points corresponding to large s values. This change reduces the excessive fractal dimension while increasing the goodness of fit and reducing the distance error. The fractal dimension, goodness of fit, and distance error results for Lai’2016 and Liu’2021 (Table 6, Table 7 and Table 8) confirm this conclusion.

In summary, the grid selection strategy based on the “consecutive integer method + weight method” can improve the goodness of fit, reduce the distance error, and obtain more accurate fractal dimension results for DBC methods. However, from the results in this section, although the grid selection strategy improves the computational accuracy compared to the original method, the calculated results of the three methods are very different. This is because many parameters affect the fractal dimension, and each method uses different optimization strategies for other parameters, resulting in many deviations in the results.

## 6. Conclusions

This study proposes an improved consecutive integer method to address the current difficulties of distorted calculation results and huge distance errors caused by the inappropriate choice of grid size s in the differential box-counting method. The synthetic FBM images, Brodatz database, and Aerials database are then used to evaluate and compare the effects of three grid size selection methods, namely the consecutive integer method, the divisor method, and the improved consecutive integer method, on the accuracy of fractal dimension calculation. Except for the different grid selections, all other parameters are identical to the DBC approaches. The results indicate that the original consecutive integer method ignores the boxes along the edges, resulting in fewer boxes to calculate. This reduces the goodness of fit and increases the distance error, distorting the estimated fractal dimension.

Although the divisor method has a small computation and can partition the whole image completely, the number of s values is too small. Then, a large amount of effective information is ignored compared with the consecutive integer method, resulting in a severe distortion of fractal dimension results and more significant distance error, and the fitting goodness is not stable. The improved strategy solves the undercounting problem of the original consecutive integer method by retaining the edge regions of images. Furthermore, the method can partition the image completely like the divisor method. Thus, the improved consecutive integer method obtains a smaller distance error than the original consecutive integer method and the divisor method, improving the accuracy of fractal dimension calculation.

## Figures and Tables

**Table 1:** Parameters for synthesizing FBM images.

Item	Value
Maxlevel	9
Sigma	4
H	0.1~0.9
Addition	1
Seed	1

**Table 2:** Fractal dimension, the goodness of fit, and distance error of three methods based on FBM images.

TFD	FD			R^2^			DE		
	ER	CI	OCI	ER	CI	OCI	ER	CI	OCI
2.1	2.0653	2.2097	2.1255	0.9979	0.9925	0.9992	0.0232	0.0045	0.0015
2.2	2.1116	2.2490	2.1654	0.9980	0.9928	0.9995	0.0230	0.0045	0.0012
2.3	2.1512	2.3030	2.2182	0.9986	0.9930	0.9995	0.0189	0.0044	0.0011
2.4	2.2171	2.3733	2.2857	0.9986	0.9931	0.9995	0.0192	0.0044	0.0011
2.5	2.2820	2.4490	2.3551	0.9985	0.9930	0.9995	0.0201	0.0045	0.0012
2.6	2.3489	2.5231	2.4243	0.9985	0.9925	0.9995	0.0203	0.0046	0.0012
2.7	2.4131	2.5939	2.4918	0.9984	0.9929	0.9995	0.0211	0.0045	0.0012
2.8	2.4727	2.6586	2.5575	0.9982	0.9940	0.9994	0.0220	0.0042	0.0013
2.9	2.5238	2.7169	2.6132	0.9980	0.9944	0.9994	0.0233	0.0040	0.0013

**Table 3:** The correlation coefficient between the calculated fractal dimension and the theoretical fractal dimension; the slope of the fitted line.

	ER	CI	OCI
γ	0.9981	0.9979	0.9983
$\hat{b}$	0.5955	0.6648	0.6355

**Table 4:** Fractal dimension, goodness of fit, and distance error of three methods based on Brodatz images.

No.	ITEM	FD			R^2^			DE		
		ER	CI	OCI	ER	CI	OCI	ER	CI	OCI
1	D1	2.5490	2.8369	2.7290	0.9933	0.9915	0.9973	0.0429	0.0050	0.0028
2	D11	2.7029	2.9103	2.8015	0.9963	0.9920	0.9988	0.0322	0.0049	0.0018
3	D21	2.8238	2.9919	2.8823	0.9976	0.9933	0.9988	0.0262	0.0045	0.0018
4	D30	2.4027	2.7106	2.6001	0.9907	0.9864	0.9954	0.0502	0.0063	0.0036
5	D41	2.6880	2.9076	2.7982	0.9957	0.9912	0.9985	0.0345	0.0051	0.0021
6	D47	2.5025	2.8229	2.7130	0.9926	0.9919	0.9962	0.0450	0.0049	0.0033
7	D53	2.7945	2.9720	2.8624	0.9967	0.9935	0.9990	0.0305	0.0044	0.0017
8	D61	2.5250	2.7589	2.6503	0.9949	0.9903	0.9982	0.0373	0.0053	0.0023
9	D65	2.6220	2.8856	2.7758	0.9931	0.9901	0.9977	0.0436	0.0054	0.0026
10	D74	2.6461	2.9308	2.8196	0.9928	0.9925	0.9975	0.0448	0.0047	0.0027
11	D81	2.7382	2.9468	2.8397	0.9968	0.9943	0.9989	0.0298	0.0041	0.0018
12	D85	2.7160	2.8882	2.7774	0.9978	0.9943	0.9993	0.0245	0.0041	0.0014
13	D91	2.3144	2.5885	2.4793	0.9928	0.9879	0.9960	0.0439	0.0059	0.0034
14	D99	2.4858	2.7552	2.6470	0.9931	0.9895	0.9974	0.0434	0.0056	0.0028
15	D104	2.8115	2.9987	2.8893	0.9966	0.9937	0.9986	0.0308	0.0043	0.0020
16	D112	2.6276	2.8853	2.7759	0.9948	0.9929	0.9982	0.0378	0.0046	0.0023

**Table 5:** Fractal dimension, goodness of fit, and distance error of three methods based on Aerials images.

No.	ITEM	FD			R^2^			DE		
		ER	CI	OCI	ER	CI	OCI	ER	CI	OCI
1	2.1.01	2.5653	2.7998	2.6939	0.9973	0.9951	0.9983	0.0272	0.0038	0.0022
2	2.1.02	2.6274	2.8440	2.7351	0.9975	0.9951	0.9982	0.0263	0.0038	0.0023
3	2.1.03	2.3861	2.5221	2.4275	0.9992	0.9968	0.9985	0.0148	0.0031	0.0021
4	2.1.04	2.5664	2.7880	2.6770	0.9981	0.9952	0.9985	0.0226	0.0038	0.0021
5	2.1.05	2.4971	2.7461	2.6369	0.9962	0.9940	0.9977	0.0323	0.0042	0.0026
6	2.1.06	2.5538	2.7413	2.6338	0.9976	0.9952	0.9982	0.0254	0.0037	0.0023
7	2.1.07	2.4586	2.6197	2.5094	0.9993	0.9929	0.9985	0.0134	0.0045	0.0021
8	2.1.08	2.4564	2.7155	2.6068	0.9977	0.9933	0.9955	0.0248	0.0044	0.0036
9	2.1.09	2.3832	2.5449	2.4448	0.9994	0.9914	0.9982	0.0132	0.0050	0.0023
10	2.1.10	2.5125	2.7052	2.5996	0.9986	0.9921	0.9976	0.0197	0.0048	0.0026
11	2.1.11	2.4268	2.6870	2.5769	0.9966	0.9943	0.9975	0.0303	0.0041	0.0027
12	2.1.12	2.4979	2.7237	2.6204	0.9984	0.9920	0.9968	0.0211	0.0048	0.0030

**Table 6:** Fractal dimension of three methods (with/without the improved strategy) based on FBM images.

TFD	Long’2013DBC	with CW	Improvement	Lai’2016DBC	with CW	Improvement	Liu’2021DBC	with CW	Improvement
2.1	2.0501	2.0935	0.0434	2.2388	2.1521	−0.0867	2.2875	2.2003	−0.0872
2.2	2.0959	2.1361	0.0402	2.2900	2.2010	−0.0889	2.3376	2.2483	−0.0893
2.3	2.1522	2.1881	0.0360	2.3288	2.2383	−0.0905	2.3722	2.2821	−0.0901
2.4	2.2161	2.2485	0.0324	2.3866	2.2944	−0.0922	2.4295	2.3366	−0.0928
2.5	2.2831	2.3141	0.0309	2.4629	2.3664	−0.0964	2.5019	2.4061	−0.0958
2.6	2.3491	2.3807	0.0316	2.5373	2.4402	−0.0971	2.5721	2.4762	−0.0959
2.7	2.4113	2.4459	0.0346	2.6084	2.5099	−0.0984	2.6420	2.5441	−0.0979
2.8	2.4669	2.5056	0.0387	2.6726	2.5739	−0.0987	2.7063	2.6080	−0.0984
2.9	2.5148	2.5575	0.0426	2.7290	2.6284	−0.1006	2.7621	2.6608	−0.1013

**Table 7:** Goodness of fit of three methods (with/without the improved strategy) based on FBM images.

TFD	Long’2013DBC	with CW	Improvement	Lai’2016DBC	with CW	Improvement	Liu’2021DBC	with CW	Improvement
2.1	0.99942	0.99969	0.00027	0.99102	0.99915	0.00813	0.99192	0.99944	0.00752
2.2	0.99940	0.99971	0.00031	0.99150	0.99898	0.00748	0.99271	0.99934	0.00663
2.3	0.99939	0.99974	0.00035	0.99275	0.99921	0.00646	0.99343	0.99938	0.00595
2.4	0.99937	0.99975	0.00039	0.99308	0.99950	0.00642	0.99374	0.99967	0.00593
2.5	0.99933	0.99975	0.00042	0.99344	0.99960	0.00617	0.99428	0.99977	0.00550
2.6	0.99927	0.99974	0.00047	0.99367	0.99955	0.00588	0.99457	0.99980	0.00523
2.7	0.99919	0.99973	0.00053	0.99373	0.99948	0.00575	0.99461	0.99982	0.00521
2.8	0.99912	0.99971	0.00059	0.99410	0.99932	0.00523	0.99492	0.99972	0.00479
2.9	0.99904	0.99969	0.00065	0.99475	0.99909	0.00434	0.99553	0.99954	0.00401

**Table 8:** Distance error of three methods (with/without the improved strategy) based on FBM images.

TFD	Long’2013DBC	with CW	Improvement	Lai’2016DBC	with CW	Improvement	Liu’2021DBC	with CW	Improvement
2.1	0.00350	0.00092	−0.00258	0.00500	0.00152	−0.00348	0.00475	0.00124	−0.00352
2.2	0.00355	0.00089	−0.00267	0.00488	0.00167	−0.00321	0.00453	0.00135	−0.00318
2.3	0.00360	0.00084	−0.00276	0.00451	0.00147	−0.00304	0.00431	0.00131	−0.00300
2.4	0.00369	0.00082	−0.00287	0.00443	0.00118	−0.00325	0.00422	0.00096	−0.00325
2.5	0.00383	0.00083	−0.00299	0.00433	0.00105	−0.00328	0.00405	0.00080	−0.00325
2.6	0.00401	0.00085	−0.00315	0.00427	0.00112	−0.00315	0.00396	0.00075	−0.00320
2.7	0.00422	0.00088	−0.00334	0.00426	0.00122	−0.00304	0.00396	0.00072	−0.00324
2.8	0.00443	0.00091	−0.00352	0.00415	0.00140	−0.00275	0.00385	0.00091	−0.00295
2.9	0.00463	0.00095	−0.00368	0.00392	0.00162	−0.00230	0.00362	0.00116	−0.00246

## Data Availability

Not applicable.

## Abbreviations

**Symbol**	**Description**
s	Size of a square grid
s_min_	The minimum value of s
s_max_	The maximum value of s
M	Image size for a square image
G	Total number of gray levels in an image
g_max_	Maximum gray level over a grid
g_min_	Minimum gray level over a grid
n_r_	Total number of boxes at scale r, required to cover the rough surface over (i, j)th grid
N_r_	Total number of boxes at scale r, required to cover the rough surface of an image
r	The scale of a square grid for an image
h	Height of a box
γ	Correlation coefficient
R^2^	Goodness of fit
$\hat{b}$	The slope of LLS fitted line (for evaluation of FBM images)
ER	Equal ratio. Indicates that s is a divisor of the image size
CI	Consecutive integer. Indicates that the value of s is all integer from s_min_ to s_max_.
CW	The “consecutive integer + weight method” strategy
OCI	Optimized consecutive integer. Indicates that the division of the *xy* plane is based on the improved consecutive integer method.
TFD	Theoretical fractal dimension refers to the fractal dimension used to generate FBM images
FD	Fractal dimension
DE	Distance error
FBM	Fractional Brownian motion
